# Computed tomography‐based radiomics prediction of CTLA4 expression and prognosis in clear cell renal cell carcinoma

**DOI:** 10.1002/cam4.5449

**Published:** 2022-11-17

**Authors:** Hongchao He, Zhijia Jin, Jun Dai, Haofei Wang, Jianqi Sun, Danfeng Xu

**Affiliations:** ^1^ Department of Urology Shanghai Ruijin Hospital, Shanghai Jiaotong University School of Medicine Shanghai China; ^2^ Department of Radiology Shanghai Ruijin Hospital, Shanghai Jiaotong University School of Medicine Shanghai China; ^3^ School of Biomedical Engineering Shanghai Jiaotong University Shanghai China

**Keywords:** biomarker, clear cell renal cell carcinoma, CTLA4, machine learning, radiomics signature

## Abstract

**Objectives:**

To predict CTLA4 expression levels and prognosis of clear cell renal cell carcinoma (ccRCC) by constructing a computed tomography‐based radiomics model and establishing a nomogram using clinicopathologic factors.

**Methods:**

The clinicopathologic parameters and genomic data were extracted from 493 ccRCC cases of the Cancer Genome Atlas (TCGA)‐KIRC database. Univariate and multivariate Cox regression and Kaplan–Meier analysis were performed for prognosis analysis. Cibersortx was applied to evaluate the immune cell composition. Radiomic features were extracted from the TCGA/the Cancer Imaging Archive (TCIA) (*n* = 102) datasets. The support vector machine (SVM) was employed to establish the radiomics signature for predicting CTLA4 expression. Receiver operating characteristic curve (ROC), decision curve analysis (DCA), and precision‐recall curve were utilized to assess the predictive performance of the radiomics signature. Correlations between radiomics score (RS) and selected features were also evaluated. An RS‐based nomogram was constructed to predict prognosis.

**Results:**

CTLA4 was significantly overexpressed in ccRCC tissues and was related to lower overall survival. A higher CTLA4 expression was independently linked to the poor prognosis (HR = 1.458, 95% CI 1.13–1.881, *p* = 0.004). The radiomics model for the prediction of CTLA4 expression levels (AUC = 0.769 in the training set, AUC = 0.724 in the validation set) was established using seven radiomic features. A significant elevation in infiltrating M2 macrophages was observed in the RS high group (*p* < 0.001). The predictive efficiencies of the RS‐based nomogram measured by AUC were 0.826 at 12 months, 0.805 at 36 months, and 0.76 at 60 months.

**Conclusions:**

CTLA4 mRNA expression status in ccRCC could be predicted noninvasively using a radiomics model based on nephrographic phase contrast‐enhanced CT images. The nomogram established by combining RS and clinicopathologic factors could predict overall survival for ccRCC patients. Our findings may help stratify prognosis of ccRCC patients and identify those who may respond best to ICI‐based treatments.

## Graphical abstract

CTLA4expression could be predicted non‐invasively using radiomics model. Overall survival of ccRCC could be predicted by a nomogram combined with radiomics score and clinicopathological factors.

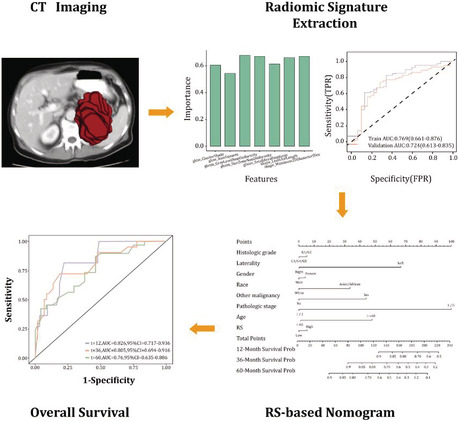

## 1 INTRODUCTION

Among all urological malignancies, renal cell carcinoma (RCC) is one of the most fatal, with high clinical incidence and increasing mortality.^1^ As a heterogeneous disease, RCC encompasses various histological subtypes, of which ccRCC is the most common one (>80% of the cases) and responsible for most mortalities. Although the 5‐year overall survival rate for surgically treated localized ccRCC is over 90%, the prognosis of advanced/metastatic ccRCC is poor, with about 10% of the 5‐year overall survival rate.^2^ However, the advent of targeted therapeutics and immunotherapies has revolutionized our treatment for ccRCC. Radical nephrectomy and nephron‐sparing surgery combined with neoadjuvant and adjuvant targeted therapy and/or immunotherapy have been demonstrated to improve prognosis in ccRCC patients. Moreover, immunotherapy is critical both as an initial therapy and as a subsequent one for antiangiogenic therapy. Previous studies indicated that different immune checkpoint inhibitors (ICIs) or a combination of ICI plus tyrosine kinase inhibitors have an established role in treating ccRCC patients.^3^, ^4^ Nevertheless, specific markers for ccRCC remain elusive, even though a wide range of biomarkers have been explored in this field.

CTLA4 is an essential immune checkpoint receptor that is expressed on activated T‐cell membranes and modulates immune responses by downregulating effector T cells and intensifying regulatory T‐cell activity.^5^, ^6^ Furthermore, there is growing evidence that CTLA4 may be a biomarker for ccRCC.^7^, ^8^ However, objective and noninvasive techniques for evaluating CTLA4 status are lacking. Radiomics, an advanced imaging analytics, collects high‐throughput data and extracts an enormous number of quantitative features from images, enabling diagnostic, prognostic, and predictive models for clinical outcomes using the imaging data from clinical routine.^9^, ^10^, ^11^ Here, we developed a radiomics signature based on enhanced CT images using the data of matched patients from the Cancer Genome Atlas (TCGA) and the Cancer Imaging Archive (TCIA) databases to predict the CTLA4 mRNA expression levels in ccRCC and constructed a nomogram supported by radiomics signature scores and clinical parameters to predict overall survival in ccRCC patients.

## 2 MATERIALS AND METHODS

### 2.1 TCGA data retrieval and analysis

Data of 537 ccRCC patients in total were collected from the TCGA database (https://portal.gdc. cancer.gov/). Cases with overall survival <30 days (*n* = 17), no genomic data (*n* = 11), unknown TNM stage and grade (*n* = 8), unknown race (*n* = 7), and bilateral tumor (*n* = 1) were excluded. Finally, a set of 493 cases was obtained according to these criteria. Detailed clinicopathologic information and genomic data were obtained. Gene expression data from ccRCC tumor samples and normal tissues were collected from the TCGA and GTEx (https://www.gtexportal.org/home/). After log2 transformation, the Toil pipeline^12^ was used to process RNAseq data in TPM format from the UCSC XENA database (https://xenabrowser.net/datapages/). The expression levels of CTLA4 were compared between normal tissues and tumor. The Kaplan–Meier method was carried out to plot survival curves. The log‐rank test was employed to analyze differences in outcomes between groups. Univariate and multivariate Cox regression using the “survival” and “forest plot” package in R was performed to identify independent risk elements for overall survival.^13^ The optimal cutoff point for CTLA4 expression was determined by the R package “survminer”^14^ and the median. The chi‐square or Fisher's exact test was conducted to assess the correlation analysis between CTLA4 expression and clinicopathologic parameters of ccRCC patients. The results were then visualized by heatmaps using the “Complex Heatmap” R package.^15^

### 2.2 Imaging data acquisition

The imaging data of all ccRCC patients (*n* = 237) were obtained from the TCIA database (http://www.cancerimagingarchive.net/). The exclusion criteria used to select images for further analysis were specified as follows: (1) non‐contrast scans, (2) postoperative scans, and (3) poor‐quality scans. Additionally, imaging data without corresponding matched clinical and genomic data in the TCGA‐KIRC cohort were also excluded, and the final integrated cohort comprising 102 ccRCC cases.

### 2.3 Image processing

Nephrographic phase contrast‐enhanced CT images were downloaded and normalized by z‐score standardization, with a mean and standard deviation of 0 and 1, respectively. Three‐dimensional slicer software was used for segmentation. To include the entire tumor, the 3D regions of interest were manually outlined on all slices of the nephrographic phase under double‐blind conditions by a urologist and a radiologist, both of whom have >8 years of work experience.

### 2.4 Radiomic features extraction and selection

An open‐source PyRadionics software version 2.1 (https://pyradiomics.readthedocs.io/en/latest/) was utilized for radiomic feature extraction. The categories of radiomic features consisted of 18 first‐order statistical features, 14 shape‐based features (2D and 3D), 16 gray‐level run‐length matrix (GLRLM) features, 24 gray‐level cooccurrence matrix (GLCM) features, 14 gray‐level dependence matrix (GLDM) features, 16 gray level size zone matrix (GLSZM) features, and 5 neighborhood gray‐tone difference matrix (NGTDM) features.

**FIGURE 1:**
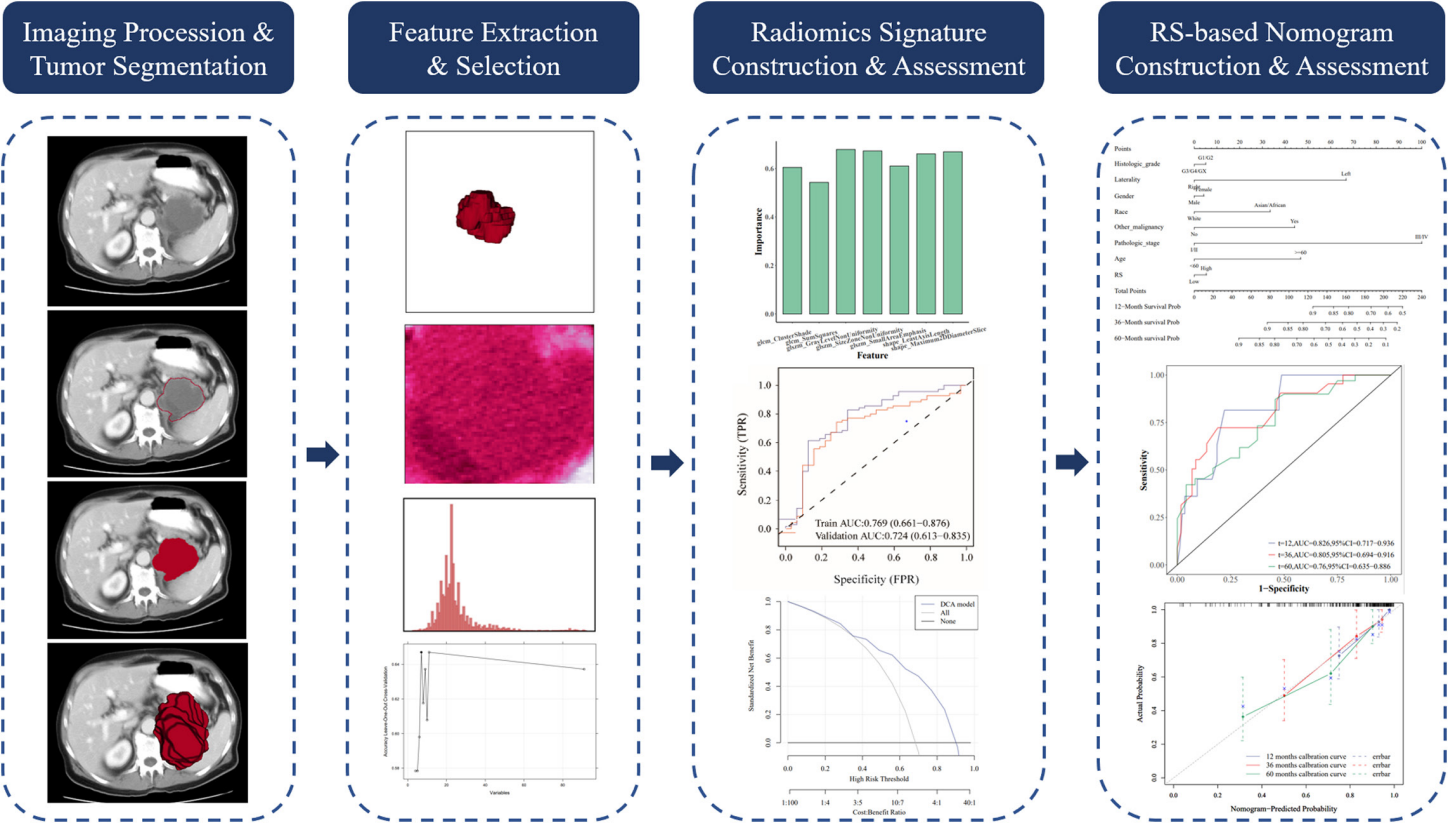
Flowchart of the radiomics process.

By using the R package “irr,” the intraclass correlation coefficient (ICC) was applied to evaluate the stability of the features,^16^ with ICC ≥0.8 indicating high consistency. Only features with ICC ≥0.80 (*n* = 91) were considered stable and selected for further analysis. The radiomics workflow is displayed in Figure 1.

### 2.5 Prediction models of CTLA4 expression and assessment

The prediction model was trained on the initial feature set and weight was assigned to each feature. Next, features with the lowest absolute weights were recursively removed from the current feature set until the required number of features was reached. To minimize overfitting, the most valuable features from the primary feature set were selected via recursive feature elimination (RFE), a method for reducing the number of features used in a classification algorithm. Also, several machine learning methods, including random forest, logistic regression, deep neural network, and support vector machine (SVM), have been applied to select features.^17^ SVM is a supervised machine learning method that can calculate decision boundaries in a feature space. Thus, SVM was performed to sort the most predictive features and finally created an optimal radiomics signature for predicting CTLA4 expression. The processes mentioned above were implemented with the “caret” and “ggpubr” packages in R.^18^, ^19^

The radiomics signature model was validated and evaluated using 10‐fold internal cross‐validation on both the training set and the validation set. First, the dataset was randomly divided into 10 groups of roughly equal size; then one of the folds was selected to be the holdout set and fit the model on the remaining nine‐folds; this process was repeated nine times using a different set each time as the holdout one. The receiver operating characteristic curve (ROC) and the area under the ROC (AUC) were calculated to assess the overall performance using the R package “pROC”.^20^ The accuracy, specificity, sensitivity, negative predictive value (NPV), and positive predictive value (PPV) were also evaluated. The precision‐recall curve was estimated using the “modEvA” R package.^21^ Model fit was assessed by the Hosmer–Lemeshow goodness‐of‐fit test using the “Resource Selection” R package. The clinical application value of the radiomics signature was evaluated by the decision curve analysis (DCA) using the “rmda” R package.^22^ The radiomics score (RS) generated according to the radiomics signature was compared between the groups with high and low CTLA4 expression using the Wilcoxon rank sum test. The Kaplan–Meier estimates and log‐rank test were used to compare the survival curves between the RS high and low groups. Landmark analysis of overall survival was performed at 36 months using the “jskm” R package. The correlation between RS and clinicopathologic parameters was assessed using the Chi‐square or Fisher's exact test.

### 2.6 Nomogram construction and assessment

The RS‐based nomogram was constructed with “rms” R package^23^ to predict the survival probability at the predefined time points (12, 36, and 60 months) according to the independent prognostic variables determined by univariate and multivariate cox regression analysis. A ROC curve with time dependence was calculated to assess the predictive ability of the nomogram at each specified time point. A calibration curve was drawn to evaluate the calibration ability of the nomogram. The clinical applicability of the RS‐based nomogram was evaluated using DCA.

### 2.7 Functional pathway analysis

To gain insight into the molecular mechanism associated with CTLA4 expression differences, the Gene Set Enrichment Analysis (GSEA) was carried out using the “cluster Profiler” R package^24^ with the Kyoto Encyclopedia of Genes, the Genomes (KEGG) pathway gene sets (c2.cp.kegg.v7.5.1.symbols.gmt), and the gene ontology (GO) term gene sets (c5.go.bp.v7.5.1.symbols.gmt, c5.go.cc.v7.5.1.symbols.gmt, and c5.go.mf.v7.5.1.symbols.gmt), respectively.

### 2.8 Immunocytes infiltration analysis

To estimate the relative immune cell infiltration levels of 22 cell types, a matrix of gene expression data from ccRCC samples were uploaded and the Cibersortx software (https://cibersortx.stanford.edu/) was used. The association between CTLA4 expression and immune cell infiltration in ccRCC was analyzed using the “limma” R package.^25^ The correlation of RS with immune cell infiltration was also evaluated.

### 2.9 Statistical analysis

Student's *t*‐test or Wilcoxon rank sum test was carried out for continuous variables and chi‐square test or Fisher's exact test was applied for categorical variables to examine the difference between groups. Log‐rank tests were used to produce and compare survival curves based on the Kaplan–Meier method. Cox regression analysis was conducted by the “glmnet” and “survival” packages in R.^26^ Spearman correlation analysis was performed for correlating CTLA4 with tumor clinical characteristics and immune cell infiltration. All statistical analyses were conducted in R studio (Version 3.5.3), and *p* < 0.05 was considered statistically significant.

## 3 RESULTS

### 3.1 CTLA4 is overexpressed in ccRCC and correlated with poor prognosis

**FIGURE 2:**
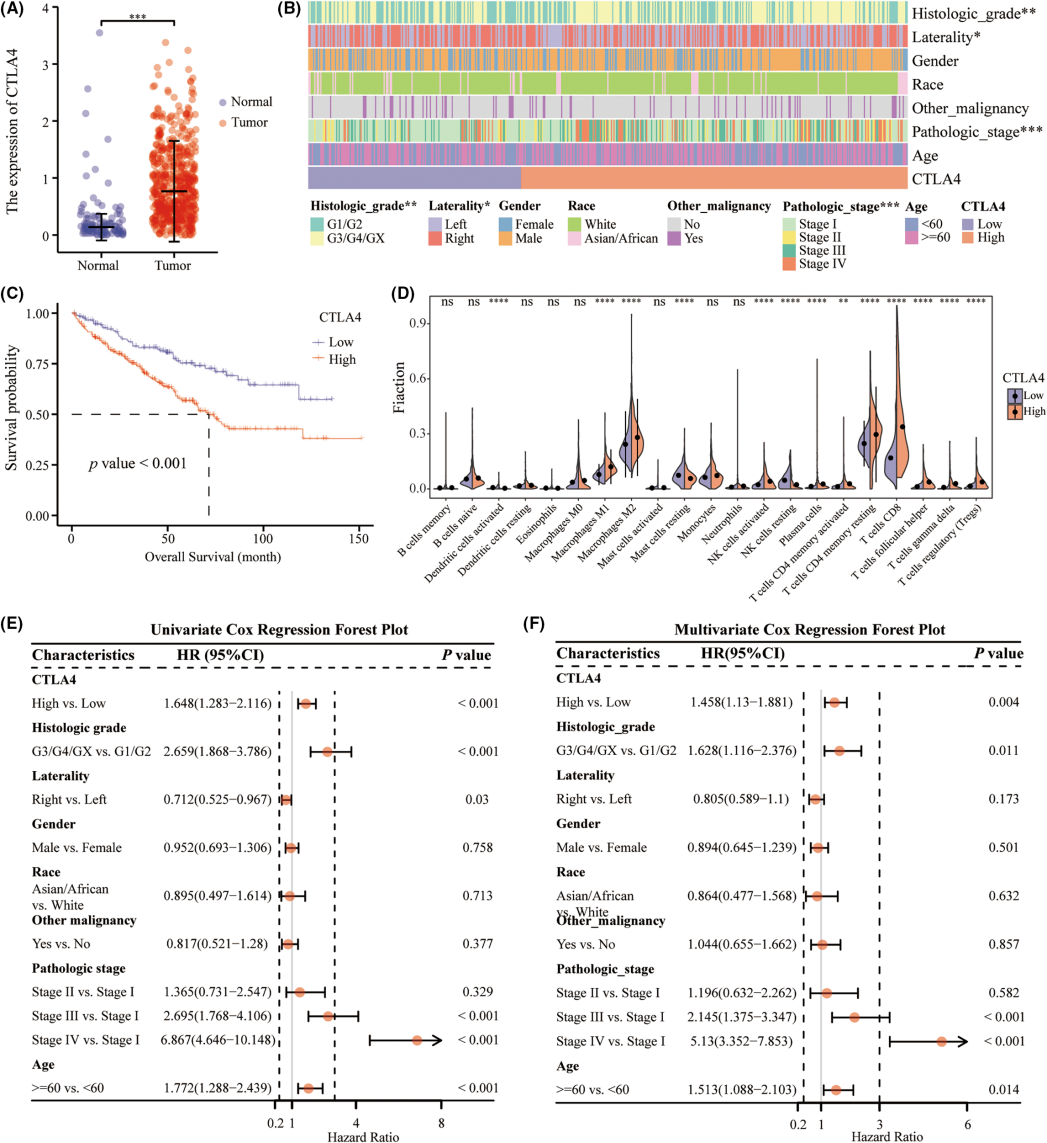
CTLA4 is overexpressed and related to poor prognosis in patients with ccRCC. (A) The expression of CTLA4 in tumor and normal tissues in the TCGA‐KIRC cohort. (B) Heatmap of the association between CTLA4 mRNA expression and clinicopathological features. (C) Kaplan–Meier analysis of overall survival in the TCGA‐KIRC cohort stratified by CTLA4 expression. (D) Violin plots of the correlation between CTLA4 expression and tumor‐infiltrating immune cells. Univariate (E) and Multivariate (F) analyses of the TCGA‐KIRC cohort.

TCGA‐KIRC cohorts were used to investigate CTLA4 expression in adjacent non‐tumor tissues and ccRCC tissues. The results suggested that the CTLA4 mRNA expression level in ccRCC tissues was notably higher than that in normal tissues (Figure 2A). The optimal cutoff point for CTLA4 expression determined by the R package “survminer was 0.4200767, while the one determined by the median was 0.633. Therefore, 493 cases from the TCGA‐KIRC cohorts enrolled in survival analyses were stratified into high (*n* = 318) and low (*n* = 175) CTLA4 expression groups using the CTLA4 expression level of 0.4200767 as an optimized cutoff. The demographic and clinical features of the patients are shown in Table 1.

**TABLE 1:** Comparison of the clinicopathological characteristics between groups with high and low CTLA4

Variables	Total (*n* = 493)	High (*n* = 318)	Low (*n* = 175)	*p* value
Histologic grade, *n* (%)				0.005
G1/G2	219 (44)	126 (40)	93 (53)	
G3/G4/GX	274 (56)	192 (60)	82 (47)	
Laterality, *n* (%)				0.056
Left	230 (47)	159 (50)	71 (41)	
Right	263 (53)	159 (50)	104 (59)	
Gender, *n* (%)				0.334
Female	168 (34)	103 (32)	65 (37)	
Male	325 (66)	215 (68)	110 (63)	
Race, *n* (%)				0.263
White	440 (89)	288 (91)	152 (87)	
Asian/African	53 (11)	30 (9)	23 (13)	
Other malignancy, *n* (%)				0.377
No	422 (86)	276 (87)	146 (83)	
Yes	71 (14)	42 (13)	29 (17)	
Pathologic stage, *n* (%)				<0.001
Stage I	246 (50)	138 (43)	108 (62)	
Stage II	52 (11)	35 (11)	17 (10)	
Stage III	113 (23)	81 (25)	32 (18)	
Stage IV	82 (17)	64 (20)	18 (10)	
Age, *n* (%)				0.403
<60	234 (47)	146 (46)	88 (50)	
> = 60	259 (53)	172 (54)	87 (50)	

The histologic grade and pathologic stage were observed to be prominently different between the groups with high and low CTLA4 expression (*p* < 0.05). In addition to this, no other significant differences were found between the two groups. The clinicopathologic characteristics of the analysis cohort were visualized by heatmaps according to CTLA4 status (Figure 2B). The results suggested that a higher CTLA4mRNA level was correlated with higher histologic grade and pathologic stage. Additionally, Kaplan–Meier survival curve analyses showed that overall survival was remarkably better in the CTLA4 low expression group than in the CTLA4 high expression group in ccRCC patients (Figure 2C, Figure S1), indicating that CTLA4 expression was associated with poor prognosis in ccRCC. Also, univariate cox regression analysis demonstrated that CTLA4 expression level was dramatically related to overall survival (HR = 1.648, 95% CI 1.283–2.116, *p* < 0.001; Figure 2E). Further multivariate cox regression analysis revealed that high CTLA4 expression was an independent prognostic factor for poor survival in ccRCC patients (HR = 1.458, 95% CI 1.13–1.881, *p* = 0.004; Figure 2F).

### 3.2 GESA analysis

**FIGURE 3:**
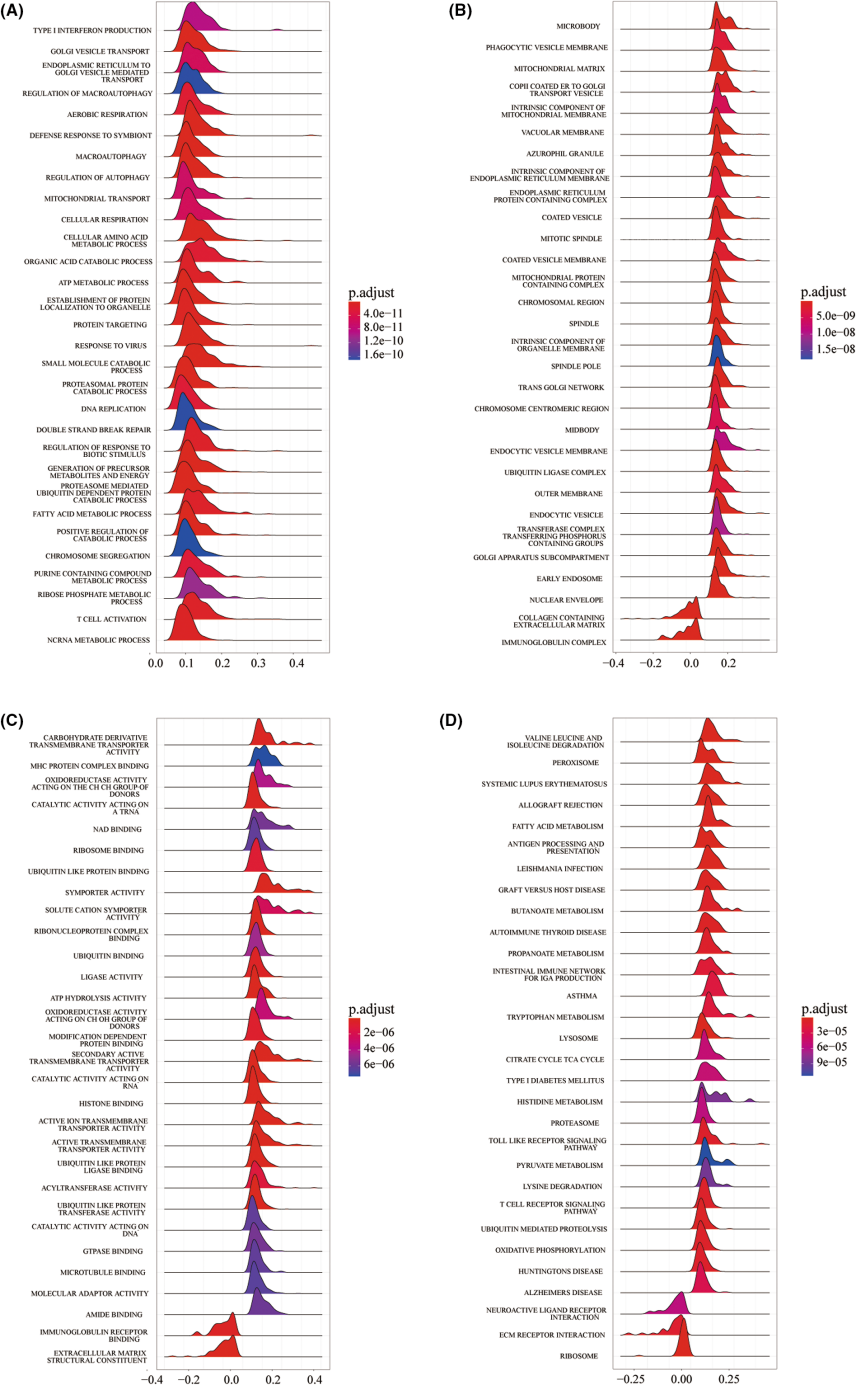
GESA‐based GO annotation and KEGG pathway enrichment analyses. (A) biological process, (B) cellular component, (C) molecular function, and (D) KEGG analysis.

GESA‐based GO annotation and enrichment analyses of the KEGG pathway were performed between groups with high and low CTLA4 expression. The top 30 significantly enriched GO terms in the categories of biological process and cellular component as well as molecular function are displayed in Figure 3. The genes in the GO terms of biological process category were predominantly involved in proteasomal protein catabolic process (483 genes; GO:0010498), autophagy regulation (333 genes; GO:0010506), and macroautophagy (304 genes; GO:0016236). The results suggested that autophagy regulation played an important role in ccRCC. The detailed genes linked to the indicated function are presented in an additional file (Table S1). KEGG analysis showed pathways enriched within each cluster at a false discovery rate (FDR) < 0.05, indicating that genes related to CTLA4 high expression were significantly enriched the gene sets associated with antigen processing and presentation, the Toll‐like receptor signaling pathway, and the T‐cell receptor signaling pathway.

### 3.3 Immune infiltration analysis

To estimate the infiltrating immune cells in ccRCC, the CIBERSORT x algorithm was applied. The results suggested that the CTLA4 high expression group had significantly elevated infiltrating of M2 macrophages, M1 macrophages, activated and resting NK cells, resting CD4^+^ memory T cells, CD8^+^ T cells, and regulatory T (Treg) cells compared to the CTLA4 low expression group (Figure 2D).

### 3.4 Feature extraction/selection and radiomics signature construction for the prediction of CTLA4 expression

**FIGURE 4:**
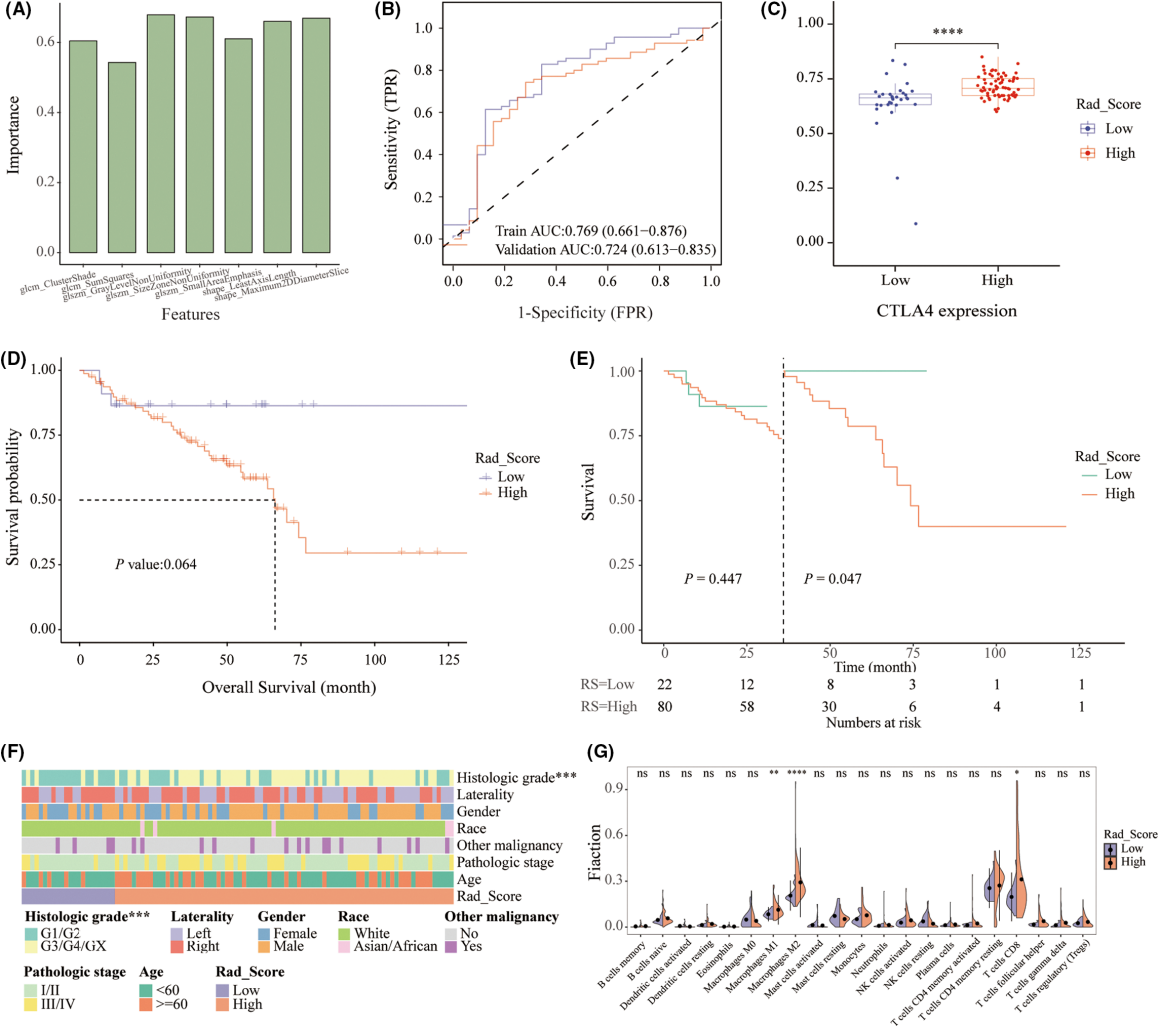
Establishment and assessment of radiomics signature to predict CTLA4 expression. (A) Features selected for model development and their importance. (B) ROC curves and AUC values for radiomics signature. (C) RS in groups with high and low CTLA4 expression. Kaplan–Meier (D) and landmark (E) analyses of overall survival stratified by RS. (F) Heatmap of the association between RS and clinicopathological features. (G) Violin plots of the correlation between RS and tumor‐infiltrating immune cells.

The radiomics analysis contained 107 features extracted from the segmented pretreatment CT images, of which 91 features with an ICC≥0.80 were regarded as robust and selected for further analysis (median ICC of 0.96). After the establishment of the radiomics CTLA4 prediction model via the SVM algorithm, seven features were finally selected to construct the radiomics signature for CTLA4 assessment through the REF algorithm. The selected features were GLCM‐cluster shade, SHAPE‐maximum 2D diameter slice, GLSZM‐size zone non‐uniformity, SHAPE‐least axis length, GLSZM‐gray level nonuniformity, GLCM‐sum squares, and GLSZM‐small area emphasis, and their importance is shown in Figure 4A.

As shown in Figure 4, the prediction of CTLA4 expression using the above radiomics signature exhibited a favorable assessment efficacy, with an AUC of 0.769 and 0.724 in the training set and the validation set, respectively. The accuracy, sensitivity, specificity, PPV, and NPV in the training and validation sets are displayed in Table 2. Furthermore, the Hosmer–Lemeshow goodness‐of‐fit tests revealed that the model was fit for the CTLA4 expression prediction (*p* = 0.079). Importantly, the DCA graphically indicated the clinical applicability of the radiomics signature.

**TABLE 2:** Predictive ability of the radiomics signature in the training set and the validation set

	Accuracy	Sensitivity	Specificity	PPV	NPV
Training set	0.696	0.614	0.875	0.915	0.509
Validation set	0.735	0.743	0.719	0.852	0.561

Abbreviations: NPV, Negative predictive value; PPV, Positive predictive value.

The RS calculated by radiomics signature represented the predicted probability of CTLA4 mRNA expression levels. The optimal cutoff point of RS was determined as 0.658 using the R package “survminer”.^14^ Next, we integrated the TCIA‐KIRC and TCGA‐KIRC datasets (102 cases were enrolled). The results showed that the RS was markedly different between the high and low CTLA4 expression groups in both the training set and the validation set, with a *p* value <0.05 (Figure 4C). The clinicopathologic characteristics of the integrated TCIA/TCGA‐KIRC cohort (*n* = 102) according to RS status are presented in Figure 4F. The results indicated that a higher RS was correlated with a higher histologic grade. Patients with high GS had a median overall survival of 66.2 months, while those with low GS did not. Although the relationship between high RS and low overall survival rate was not found by the Kaplan–Meier analysis, a crossover of survival curves was observed. Thus, the subsequent landmark analysis was performed and revealed that high RS was a risk factor in the late phase of follow‐up and that higher RS was associated with a poorer prognosis after 36 months (Figure 4D,E). In addition, the infiltrating M2 macrophages were prominently elevated in the RS high group compared to the RS low group (Figure 4G).

### 3.5 Construction of a nomogram combining radiomics with clinical risk factors

**FIGURE 5:**
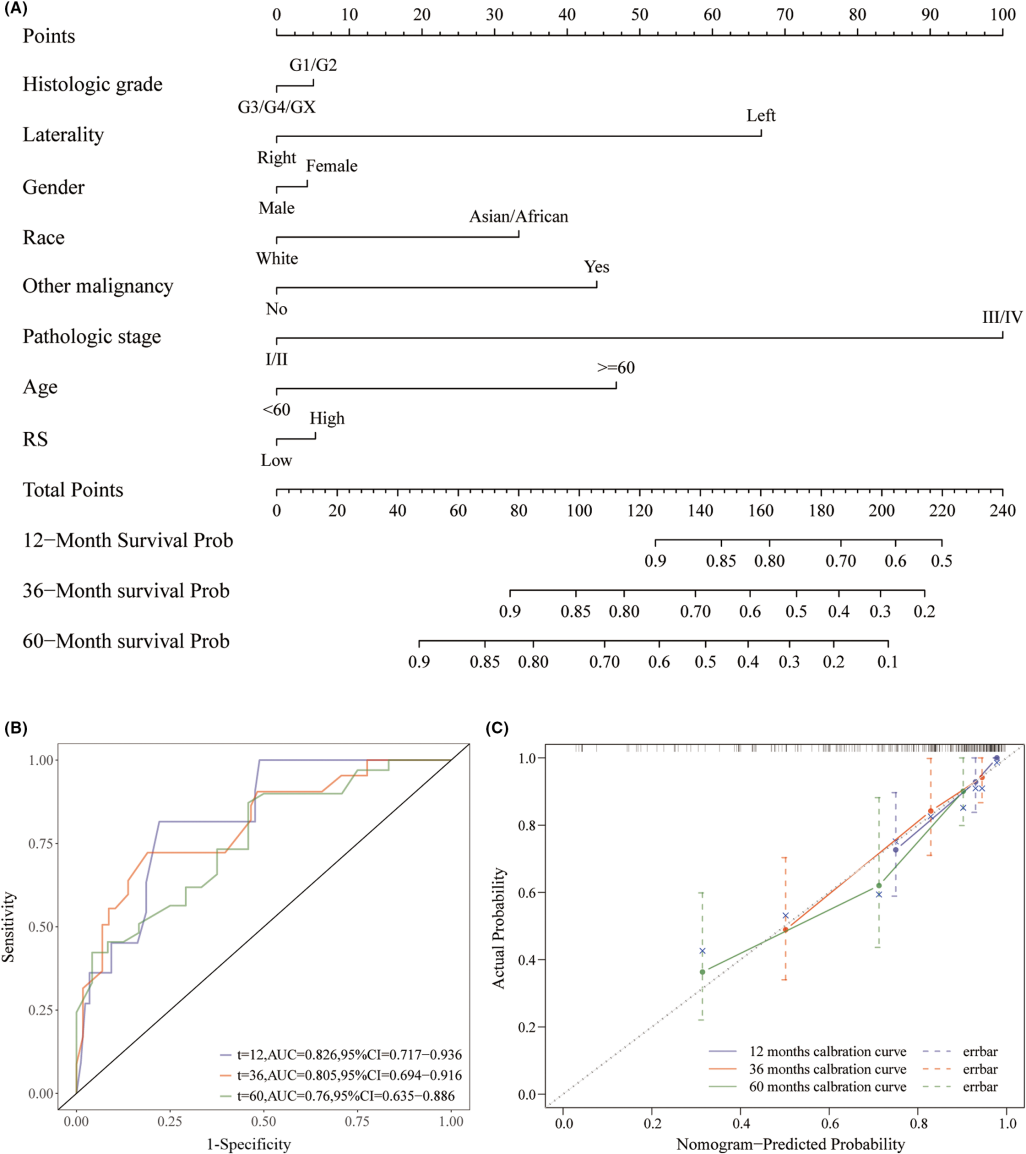
Construction and evaluation of RS‐based nomogram. (A) Nomogram used to predict the overall survival probability. (B) ROC curves and AUC values for RS‐based nomogram. (C) Calibration curve of the nomogram.

Based on the variables independently associated with prognosis from the multivariate Cox regression analysis, an RS‐based nomogram was developed to predict overall survival probability at 12, 36, and 60 months (Figure 5A). The AUC value of the RS‐based nomogram was 0.826 at 12 months, 0.805 at 36 months, and 0.76 at 60 months (Figure 5B). As displayed in Figure 5C, the calibration plot was very close to the diagonal, indicating an excellent calibration (Figure 5C). Additionally, the decision curve analysis at 12, 36, and 60 months all graphically demonstrated the clinical utility of the RS‐based nomogram.

## 4 DISCUSSION

Over the past 20 years, the treatment paradigm for ccRCC has significantly revolutionized with the emergence of targeted agents and immunotherapeutic drugs. A previous study illuminated that ICI can target and block PD‐1 or CTLA4 to restore tumor‐specific T‐cell immunity.^27^ Also, combination immune checkpoint blockade strategies (Concurrent PD‐1 and CTLA‐4 blockade using nivolumab plus ipilimumab) represent encouraging clinical benefits and have been authorized for the first‐line treatment of advanced/metastatic patients with ccRCC.^28^, ^29^ Earlier research demonstrated that immune checkpoint molecules (e.g., PD‐L1, LAG3) are involved in diverse biological processes and may serve as promising biomarkers for stratified prognosis or as excellent biomarkers for predicting the response to immune treatment in advanced urothelial carcinoma,^30^ colorectal cancer,^31^ and non‐small cell lung cancer.^32^ However, no reliable and generally accepted markers have been established in ccRCC until recently. There was evidence that PD‐L1 may be a measure of poor prognosis but does not entirely predict ICI‐based combination response.^33^, ^34^

CTLA4 is an immune checkpoint molecule and a transmembrane protein expressed by regulatory T cells and activated T cells, which delivers inhibitory signals to T‐cell activation. Sharing a significant sequence identity with a homologous receptor CD28, CTLA4 has a higher affinity for the costimulatory ligands CD80 and CD86 on antigen‐presenting cells.^35^ At the same time, the role of CTLA4 in ccRCC has attracted growing emphasis. A pan‐cancer analysis indicated that CTLA4 has a high prognostic value as a biomarker in some cancer types, including ccRCC.^7^ Previous studies suggested that CTLA4 accelerates the progression of ccRCC and is associated with OS, progression‐free survival (PFS) and disease‐free survival (DFS) in ccRCC patients.^8^, ^36^ Furthermore, CTLA4 promoter hypomethylation is negatively correlated with CTLA4 mRNA expression, predicting the response and favorable outcome of immunotherapy in ccRCC.^37^ Consistent with earlier works, our findings also demonstrated that CTLA4 was dramatically overexpressed in ccRCC tissues compared to normal tissues, and CTLA4 was an independent risk factor for poor prognosis. Our study also observed that infiltrating M2 macrophages and regulatory T cells were notably elevated in the group with CTLA4 high expression, indicating that CTLA4 promoted tumor growth by facilitating immune escape. This was in line with the previous findings that CTLA4 could deeply affect the landscape of tumor‐infiltrating lymphocytes with an immunosuppressed phenotype.^7^, ^8^ Of course, further basic research with a more comprehensive study design is necessary to investigate the exact mechanism.

Accumulating evidence indicates that CTLA4 is a valuable biomarker for ccRCC. However, there is no available method to noninvasively assess the CTLA4 mRNA expression levels. According to a previous study, higher serum CTLA4 level was correlated with melanoma response and clinical benefit from ipilimumab therapy, but the work is greatly limited due to a very small sample size.^38^ Currently, the only method to determine the CTLA4 mRNA expression in the tumor is by invasive biopsy or surgical resection. Nonetheless, the biopsy is limited to a small part of the tumor and cannot fully represent the CTLA4 level in the entire tumor, and surgery is not suitable for advanced ccRCC. Radiomics, which transforms medical imagines into an enormous number of quantitative descriptors of oncologic tissues using cutting‐edge computational approaches, has been applied for the differentiation diagnosis^39^ and recurrence/metastasis risk^40^ and survival prediction in ccRCC.^41^ In this study, a radiomics model was built to decode CTLA4 mRNA expression levels and was proved to be effective and clinically useful. Moreover, we constructed an RS‐based nomogram that could be utilized to predict overall survival in ccRCC patients. Our results demonstrated that the expression status of CTLA4 could be accessed noninvasively from radiomics analysis of CT imaging, which might affect clinical decision‐making.

Despite these novel findings, this study has some limitations that deserve comment. First, the conclusion of this study was based on the data obtained from public databases, which was inevitably limited by the quality of data and images. Second, the number of total ccRCC cases (*n* = 102) and advanced/metastatic ccRCC cases (*n* = 37) recruited in this study were relatively small. Moreover, it remains unclear whether the established radiomics signature and the RS‐based nomogram can be applied to advanced/metastatic ccRCC with adequate predictive value. Therefore, there is an urgent need to further explore a larger number of ccRCC patients with different disease stages (localized, locally advanced, and advanced/metastatic).

In conclusion, we identified an effective and stable radiomics model based on contrast‐enhanced CT images for determining CTLA4 mRNA expression status and combined RS and clinicopathologic factors to create a nomogram for predicting overall survival in patients with ccRCC. All these findings may help stratify prognosis and identify patients who are likely to respond best to ICI‐based treatments.

## AUTHOR CONTRIBUTIONS

**Hongchao HE:** Conceptualization (equal); data curation (equal); formal analysis (equal); writing – original draft (equal). **Zhijia Jin:** Data curation (equal); formal analysis (equal). **Jun Dai:** Data curation (equal); formal analysis (equal). **Haofei Wang:** Visualization (equal). **Jianqi Sun:** Visualization (equal). **Danfeng Xu:** Conceptualization (equal); writing – review and editing (equal).

## FUNDING INFORMATION

This study was supported by the Medical and Technology Intercrossing Research Foundation of Shanghai Jiaotong University (YG2016QN65) and the Guangci Excellent Youth Program of Ruijin Hospital (GCQN‐2018‐B15).

## CONFLICT OF INTERESTS

The authors declare that the research was conducted in the absence of any commercial or financial relationships that could be construed as a potential conflict of interest.

## ETHICAL STATEMENTS

All data were collected from the TCGA database (https://portal.gdc.cancer.gov/) and TCIA database (http://www.cancerimagingarchive.net/). TCGA and TCIA belong to public databases. The patients involved in the database have obtained ethical approval. Users can download relevant data free for research and publication of relevant articles. Our study is based on open‐source data, so there are no ethical issues and other conflict of interests.

## Supporting information


Figure S1.

Supplemental Table S1.

Supplemental Table S2.

## Data Availability

All data were collected from the TCGA database (https://portal.gdc.cancer.gov/) and the TCIA database (http://www.cancerimagingarchive.net/). Users can download relevant data free for research and publication of relevant articles. The data supporting the conclusions of this manuscript will be made available by the authors, without reservation, to any qualified researcher upon request.
