# Supplementary figures and images for: Computed tomography‐based radiomics prediction of CTLA4 expression and prognosis in clear cell renal cell carcinoma
Source: Cancer Med. 2022 Nov 17;12(6):7627–38. doi: 10.1002/cam4.5449 (PMC10067074; doi:10.1002/cam4.5449)

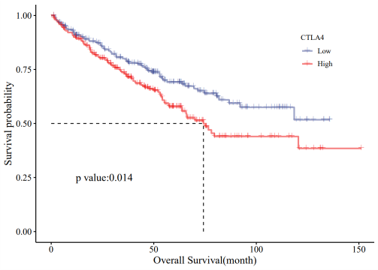

Supplement: Supplementary file 1 — Figure S1. [file CAM4-12-7627-s001.tif]
