# Supplementary material for: Computed tomography‐based radiomics prediction of CTLA4 expression and prognosis in clear cell renal cell carcinoma
Source: Cancer Med. 2022 Nov 17;12(6):7627–38. doi: 10.1002/cam4.5449 (PMC10067074; doi:10.1002/cam4.5449)
Supplement: Supplementary file 3 — Supplemental Table S2. [file CAM4-12-7627-s002.docx]

Supplemental Table 2. Results of cox regression analysis in TCIA dataset

|  | Univariate analysis | | Multivariate analysis | |
| --- | --- | --- | --- | --- |
|  |  | P value |  | P value |
| Histologic_grade: G3/G4/GX vs. G1/G2 | 2.221(1.041-4.738) | 0.039 | 0.905(0.373-2.193) | 0.824 |
| Laterality: Right vs. Left | 0.479(0.244-0.938) | 0.032 | 0.265(0.114-0.615) | 0.002 |
| Gender: Male vs. Female | 0.762(0.392-1.482) | 0.423 | 0.92(0.43-1.966) | 0.829 |
| Race: Asian/African vs. White | 1.381(0.327-5.831) | 0.66 | 1.94(0.398-9.446) | 0.412 |
| Other_malignancy: Yes vs. No | 1.386(0.63-3.049) | 0.417 | 2.403(0.847-6.818) | 0.1 |
| Pathologic_stage: III/IV vs. I/II | 3.804(1.919-7.538) | < 0.001 | 7.31(2.827-18.904) | < 0.001 |
| Age: >=60 vs. <60 | 1.789(0.926-3.457) | 0.084 | 2.535(1.192-5.39) | 0.016 |
| RS: High vs. Low | 2.904(0.889-9.481) | 0.077 | 1.112(0.288-4.284) | 0.878 |
